# Targeting CD74 in multiple myeloma with the novel, site-specific antibody-drug conjugate STRO-001

**DOI:** 10.18632/oncotarget.26491

**Published:** 2018-12-28

**Authors:** Cristina L. Abrahams, Xiaofan Li, Millicent Embry, Abigail Yu, Stellanie Krimm, Sarah Krueger, Nancy Y. Greenland, Kwun Wah Wen, Chris Jones, Venita DeAlmeida, Willy A. Solis, Shannon Matheny, Toni Kline, Alice Y. Yam, Ryan Stafford, Arun P. Wiita, Trevor Hallam, Mark Lupher, Arturo Molina

**Affiliations:** ^1^ Sutro Biopharma, Inc., South San Francisco, California, USA; ^2^ MI Bioresearch, Ann Arbor, MI, USA; ^3^ Department of Pathology and Laboratory Medicine, University of California, San Francisco, CA, USA

**Keywords:** CD74, antibody-drug conjugate, multiple myeloma, xenograft models, STRO-001

## Abstract

STRO-001 is a site-specific, predominantly single-species, fully human, aglycosylated anti-CD74 antibody-drug conjugate incorporating a non-cleavable linker-maytansinoid warhead with a drug-antibody ratio of 2 which was produced by a novel cell-free antibody synthesis platform. We examined the potential pharmacodynamics and anti-tumor effects of STRO-001 in multiple myeloma (MM). CD74 expression was assessed in MM cell lines and primary bone marrow (BM) MM biopsies. CD74 mRNA was detectable in CD138+ enriched plasma cells from 100% (892/892) of patients with newly diagnosed MM. Immunohistochemistry confirmed CD74 expression in 35/36 BM biopsies from patients with newly diagnosed and relapsed/refractory MM. Cytotoxicity assays demonstrated nanomolar STRO-001 potency in 4/6 MM cell lines. In ARP-1 and MM.1S tumor-bearing mice, repeat STRO-001 dosing provided significant antitumor activity with eradication of malignant hCD138+ BM plasma cells and prolonged survival. In a luciferase-expressing MM.1S xenograft model, dose-dependent STRO-001 efficacy was confirmed using bioluminescent imaging and BM tumor burden quantification. Consistent with the intended pharmacodynamic effect, STRO-001 induced dose-responsive, reversible B-cell and monocyte depletion in cynomolgus monkeys, up to a maximum tolerated 10 mg/kg, with no evidence of off-target toxicity. Collectively, these data suggest that STRO-001 is a promising therapeutic agent for the treatment of MM.

## INTRODUCTION

Treatment of patients with multiple myeloma (MM) with proteasome inhibitors, such as bortezomib, and immunomodulatory agents, such as lenalidomide, in frontline and maintenance settings has greatly improved survival [1, 2]. Second generation proteasome inhibitors (e.g., carfilzomib and ixazomib), immunomodulatory agents (e.g., pomalidomide), histone deacetylase inhibitors (e.g., panobinostat), and monoclonal antibodies (e.g., elotuzumab and daratumumab) have proven to be highly effective in relapsed and refractory MM, particularly when used in combination therapy [1, 3, 4]. Many patients with MM, however, ultimately relapse and die of progressive disease. In 2017 alone, it was estimated that over 30,000 people would be diagnosed with MM in the US and that 12,500 patients would die of the disease [4]. This underscores the need for new agents which are both effective and well tolerated [3, 5].

CD74, also known as HLA-DR-associated invariant chain, is a type II transmembrane glycoprotein that functions as an MHC class II chaperone and as a high affinity receptor for the pro-inflammatory cytokine macrophage migration inhibitory factor [6]. Upon binding to macrophage migration inhibitory factor, the CD74-intracellular domain translocates to the nucleus where it acts in conjunction with NF-κB pathway members to induce B-cell proliferation and survival. CD74 is over-expressed on MM and non-Hodgkin lymphoma (NHL) cell lines and various tumors, including the majority of MM patient biopsies tested, but its expression in normal tissues is limited to B cells, monocytes, macrophages, dendritic cells, Langerhans cells, activated T-cell subsets, and thymic epithelium [7–12]. This, in addition to its rapid internalization and recycling, makes it an attractive therapeutic target for MM [8, 10, 13]. After promising preclinical results, an anti-CD74 antibody, milatuzumab [11], reached clinical development for relapsed or refractory (R/R) MM. In a phase 1 study of 25 heavily pretreated patients, however, no objective responses were observed, although moderate decreases of B-cell levels and a 26% disease stabilization rate were reported [14].

Antibody-drug conjugates (ADCs) are emerging as a promising class of cancer biopharmaceuticals that combine the specificity of monoclonal antibodies with the anti-tumor activity of cytotoxic agents [15–19]. The successful development of clinically effective antibodies targeting over-expressed cell-surface proteins, such as SLAMF7 and CD38 [20] in myeloma, suggests that this approach can also be applied to the development of ADCs [6]. In the case of CD74, efforts were made to improve the clinical activity of the monoclonal antibody milatuzumab through conjugation with the anthracycline chemotherapeutic agent doxorubicin [21]. The resulting ADC, hLL1-DOX, was investigated in phase 1/2 trials in relapsed NHL and chronic lymphocytic leukemia (NCT01585688) and in MM (NCT01101594), but results have not been reported.

Here we report results for STRO-001, a novel ADC comprised of an aglycosylated anti-CD74 IgG1 human antibody (SP7219) conjugated to a non-cleavable linker-maytansinoid warhead in a site-specific manner, resulting in a predominantly single-species ADC with a drug-antibody ratio (DAR) of 2. We describe the expression and prevalence of CD74 in MM patient samples and cell lines as detected by flow cytometry and immunohistochemistry using SP7219 and CD74 mRNA analysis and report the generation and preliminary efficacy of STRO-001 in *in vitro* and *in vivo* models of MM. STRO-001 safety and pharmacodynamics in cynomolgus monkeys are also described.

## RESULTS

### Generation of STRO-001

**Figure 1:**
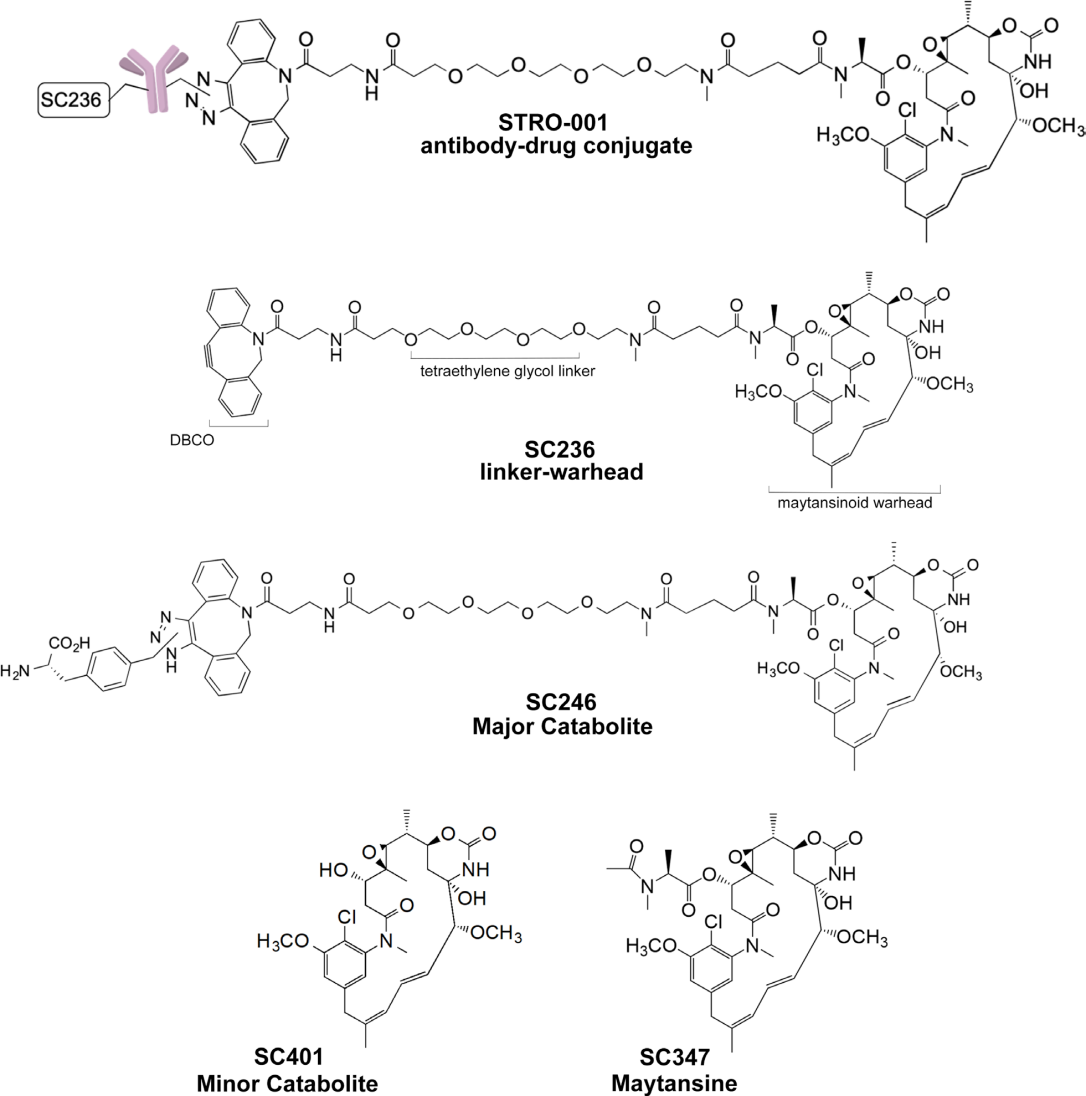
Structures of STRO-001, SC236, SC246, SC401 and SC347

**Figure 2:**
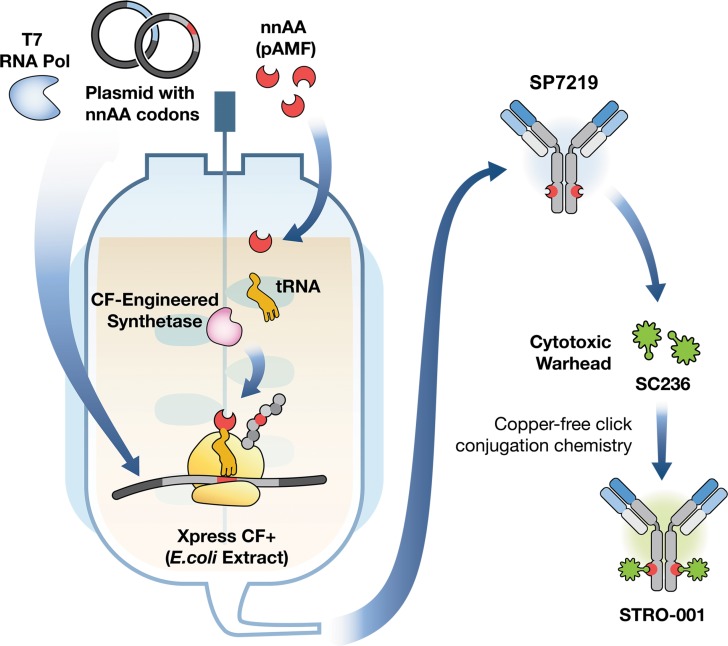
Generation of the CD74-targeting lead antibody and a novel, specific and homogeneous ADC, STRO-001 STRO-001 is a novel CD74-targeting ADC containing an anti-CD74 aglycosylated human IgG1 antibody (SP7219) conjugated to two non-cleavable DBCO-maytansinoid linker-warheads (SC236) using site-specific conjugation technology. SP7219 is generated using a cell-free protein synthesis technology called XpressCF+™. The SC236 warhead is conjugated to SP7219 at specific sites via a copper-free click conjugation chemistry, which results in a well-defined, predominantly single-molecular species drug product.

SP7219, the anti-CD74 antibody in STRO-001 (Figure 1), was discovered from a Fab-ribosome display library [22], and selected based on its optimal properties in cell binding, cell-based internalization, affinity, and stability. SP7219 was generated using Sutro's XpressCF+™ platform, a coupled *in vitro* transcription/translation system that contains the translational machinery and energy generation systems required to express proteins directly from a plasmid DNA template (Figure 2). XpressCF+™ has an engineered RF1 mutant which facilitates efficient incorporation of the non-natural amino acid *para*-azidomethyl-L-phenylalanine (pAMF) at positions designated by an amber stop codon [23]. The XpressCF+™ generates aglycosylated antibodies, which lack Fc effector function [24]. In STRO-001, the amber stop codons were directed to the F404 (EU numbering) position on each heavy chain of SP7219. STRO-001 was then produced by strain-promoted azide-alkyne cycloaddition (SPAAC) between each pAMF of SP7219 and dibenzocyclooctyne (DBCO) of SC236, a non-cleavable maytansinoid linker-warhead (Figure 1 and Supplementary Appendix). This resulted in a predominantly single-species ADC with near complete conjugation and an average DAR close to 2.

### CD74 expression in bone marrow (BM) specimens from MM patients

**Figure 3:**
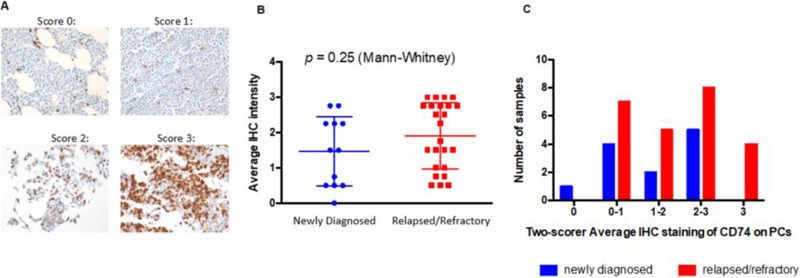
Expression of CD74 in bone marrow samples from patients with newly diagnosed and relapsed/refractory multiple myeloma (**A**) Representative IHC staining for CD74 in patients with multiple myeloma. Scores were from 0 (no staining) through 3 (strong staining) on plasma cells. 40× magnification. (**B**) Two individual pathologists blindly scored samples from newly diagnosed (blue) or relapsed/refractory (red) patients. (**C**) 35 of 36 MM patients have plasma cells expressing at least some level of CD74, underscoring its utility as a target in MM.

Primary MM patient BM core biopsies were analyzed by immunohistochemistry (IHC) using biotinylated SP7219 (Figure 3A). Twelve samples from patients with newly diagnosed MM (Figure 3B, left) and 24 samples from patients with relapsed/refractory (R/R) myeloma were analyzed (Figure 3B, right). Overall, CD74 expression levels were not significantly different (Figure 3B). Two independent pathologists, blinded as to treatment status, scored the intensity of CD74 staining at 0, 0–1, 1–2, 2–3 and 3 (Figure 3C). Of the newly diagnosed group, 1 sample scored a 0, 4 samples scored between 0–1, 2 samples were between 1–2, and 5 were between 2–3. By contrast, all samples from the R/R group expressed some level of CD74, with 7 scoring between 0–1, 5 between 1–2, 8 between 2–3, and 4 samples scoring 3. Of the 36 samples analyzed, only 1 sample did not express any CD74 and the majority (67% of R/R samples) expressed CD74 levels higher than 1.

### CD74 mRNA expression in plasma cells from MM patients

**Figure 4:**
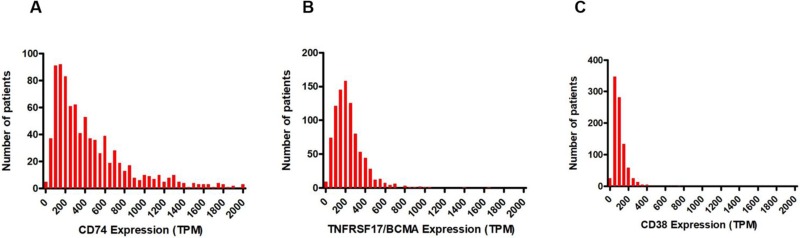
Frequencies of mRNA expression in TPM in CD138+ enriched plasma cells from 892 patients in the MMRF CoMMpass study (**A**) CD74. (**B**) BCMA. (**C**) CD38.

Analysis of data from the Multiple Myeloma Research Foundation (MMRF) Relating Clinical Outcomes in Multiple Myeloma to Personal Assessment of Genetic Profile (CoMMpass) study [25] found that CD74 transcript was expressed at detectable levels (transcripts per million [TPM] >1) in CD138+ enriched plasma cells from all 892 newly-diagnosed MM patients included in the study (Figure 4A). Furthermore, a comparison to the well-validated MM surface targets CD38 and BCMA (*TNRFSF17*) revealed that CD74 had the highest mRNA expression of the 3 (Figure 4B, 4C). Transcriptome analysis also suggested that 10.9% of patient tumors (97 of 892) may express extremely high levels of CD74 (TPM >1000). These results further validate the IHC analysis results demonstrating consistent CD74 protein expression in malignant plasma cells from patients with MM.

### CD74 expression and cytotoxicity of STRO-001 in MM cell lines

**Figure 5:**
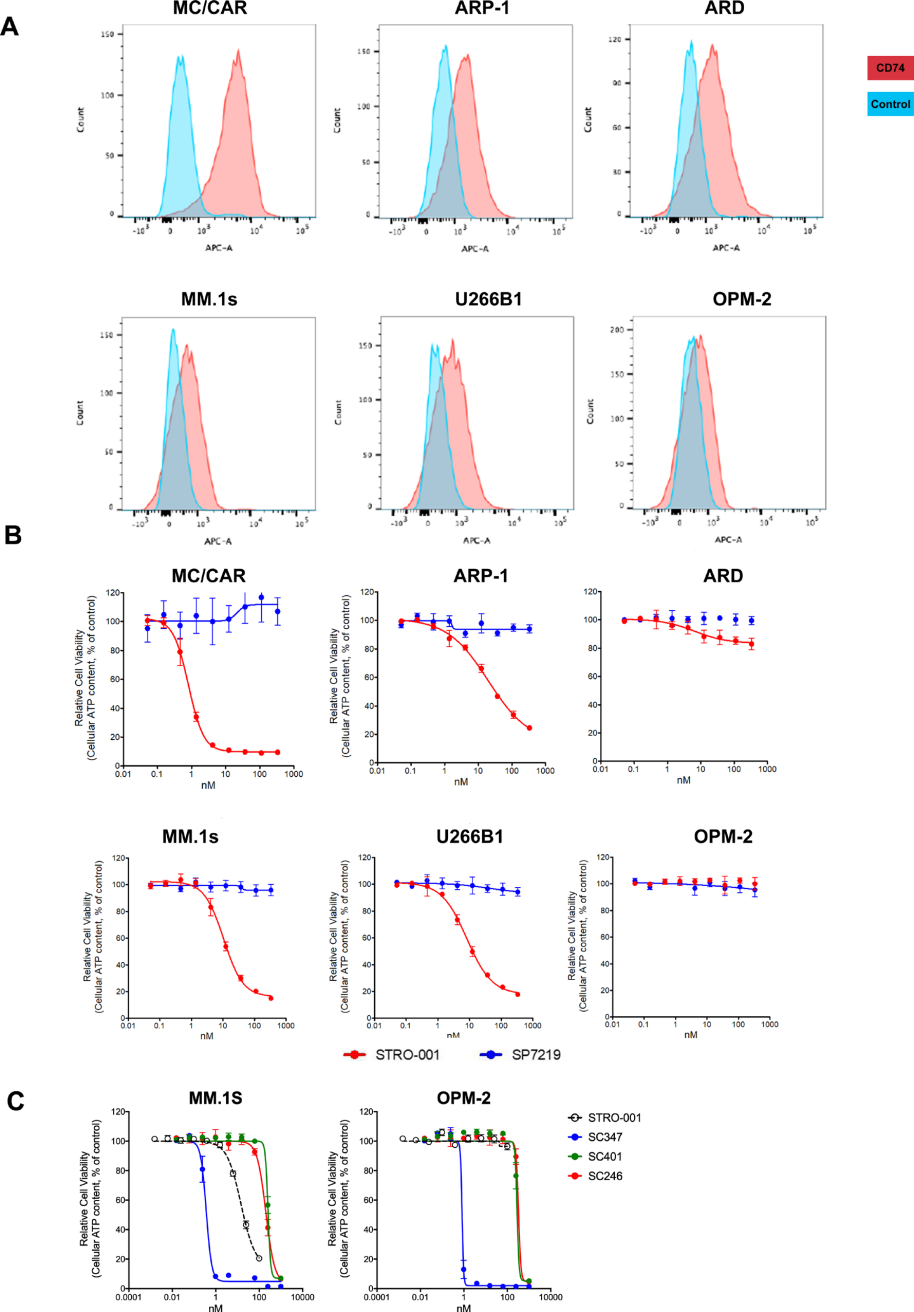
Expression of CD74 in multiple myeloma cell lines and cytotoxicity of STRO-001 and catabolites (**A**) Multiple myeloma cell lines were evaluated for expression of CD74 using Alexa647-conjugated SP7219. Unstained cells were used as negative controls. (**B**) The ADC, STRO-001, was used to determine the EC50 and percent span of killing in these MM cell lines, while the unconjugated antibody SP7219 did not show any activity in these cell lines. (**C**) Cytotoxicities of catabolites SC401 and SC246 were compared with STRO-001 and reference maytansine SC347 in CD74-expressing (MM.1S) and non-expressing (OPM-2) cells.

We next tested the cell binding and cell killing activities of STRO-001 in MM cell lines. Of the 6 cell lines tested, MC/CAR cells demonstrated the highest level of CD74 expression on the cell surface (Figure 5A and Table 1). CD74 surface copy number on ARP-1, ARD, MM.1S, U266B1 and OPM-2 cells were near or below the detection limit of the antibody binding capacity (ABC) assay, albeit all had positive binding by mean fluorescence intensity (MFI). Interestingly, STRO-001 showed potent cell killing activity, with EC50 in the sub-nM to nM range for ARP-1, MM1.S and U266B1 cells despite the relatively low CD74 expression on these cells (Figures 5A and 5B). SP7219, the unconjugated antibody, was not cytotoxic in these cells (Figure 5B). On MC/CAR, ARP-1, U266B1 and MM1.S cells, STRO-001 cell killing activity roughly correlated with CD74 expression on the cell surface with higher expression of CD74 correlating with better cell killing activity (lower EC50).

**Table 1:** STRO-001 cytotoxicity in multiple myeloma cell lines

	SP7219 cell binding activity		STRO-001 cell killing activity	
Multiple myeloma cell line	MFI	CD74 copies/cell (determined by ABC assay)	EC50 (nM)	Percentage killing
MC/CAR	3744	42,981	0.84	90
ARP-1	924	9523	23	88
ARD	841	8605	NC	NC
U266B1	427	<8445	8.8	83
MM.1S	332	<8445	12	85
OPM-2	218	<8445	NK	NK

ABC, antibody binding capacity; MFI, geometric median fluorescence intensity; NC, not calculable; NK, no killing.

Parallel studies performed *in vitro* and *in vivo* indicated that STRO-001 is processed within the cells, resulting in release of two catabolites: a major catabolite, SC246, and a minor catabolite, SC401 (Figure 1) (data on file, Sutro BioPharma). CD74 antigen expressing (MM.1S) and non-expressing (OPM-2) cells were exposed to SC246, SC401, and a reference maytansine benchmark compound (SC347; Figure 5C). SC246 and SC401 proved to be approximately 300–500 times less cytotoxic than SC347 and >15 times less cytotoxic than STRO-001 in these assays. Their reduced cytotoxicity upon extracellular exposure (relative to intracellular generation from STRO-001 within target cells) may benefit the safety profile of STRO-001 through reduction of non-target-associated toxicity.

### STRO-001 exhibits potent anti-tumor activity in disseminated MM models

**Figure 6:**
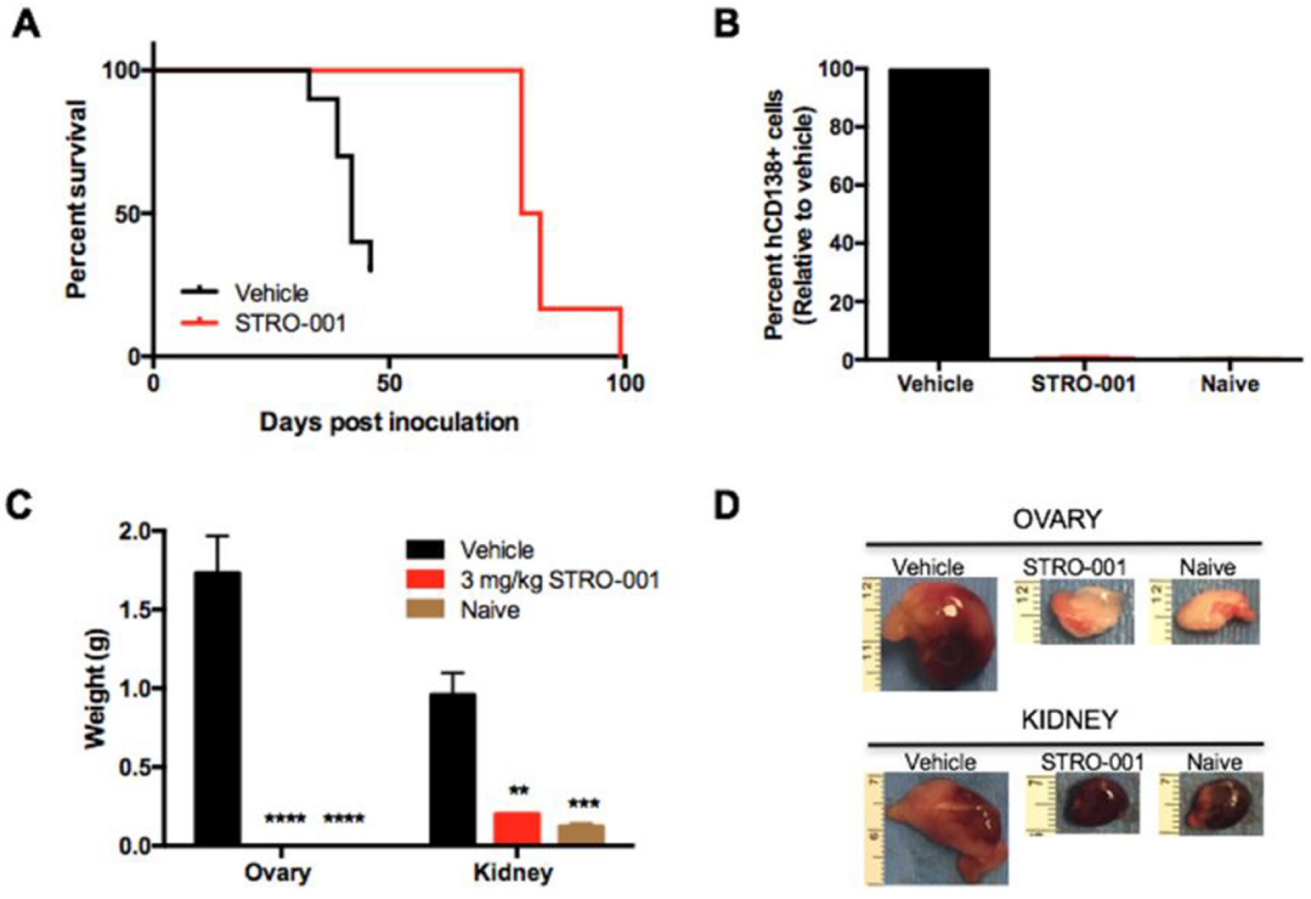
STRO-001 significantly reduces tumor burden in the ARP-1 myeloma model SCID mice were inoculated with ARP-1 MM tumor cells by intravenous injection. Mice were treated weekly with 3 mg/kg STRO-001 beginning at 14 days post-tumor inoculation for one month. Naïve control mice were not inoculated with tumor cells and did not receive treatment. (**A**) Kaplan-Meier survival curves in response to treatment. (**B**) Quantification of hCD138+ cells in the bone marrow depicted as percent relative to vehicle control (set as 100% maximum) on day 49. (**C**, **D**) Quantification of weights and representative images of internal visceral tumors in and around the ovary and kidney on day 49. Graphs are shown as average values ± SEM.

The *in vivo* efficacy of STRO-001 was examined in the disseminated MM ARP-1 model which engrafts in the BM and other internal organs. Tumor-bearing animals were treated 14 days post-tumor inoculation with a repeat dosing regimen of vehicle or 3 mg/kg STRO-001 once a week for 4 weeks. Kaplan–Meier survival curves were generated using body weight change defined as greater than 9% increase (associated with development of internal abdominal tumors) and moribundity. Mean survival of the vehicle-treated group was 40 days, while STRO-001 treatment prolonged survival to 79 days (Figure 6A). Tumor burden within the BM was evaluated by staining for hCD138+ cells on the day of sacrifice using flow cytometry. Repeat dosing with 3 mg/kg STRO-001 resulted in marked reduction in BM tumor burden compared with vehicle-treated animals and exhibited similar hCD138+ profile as naïve controls on day 49 (Figure 6B). Tumor burden was also evaluated by assessing the weight of affected internal organs. In vehicle-treated animals, ARP-1 tumors developed around the kidneys and ovaries with mean weights at 1.0 and 1.7 grams, respectively (Figure 6C). In contrast, kidneys and ovaries from STRO-001-treated animals were significantly smaller compared to vehicle control and presented comparable weights to organs harvested from naïve control mice on day 49 (Figures 6C and 6D).

**Figure 7:**
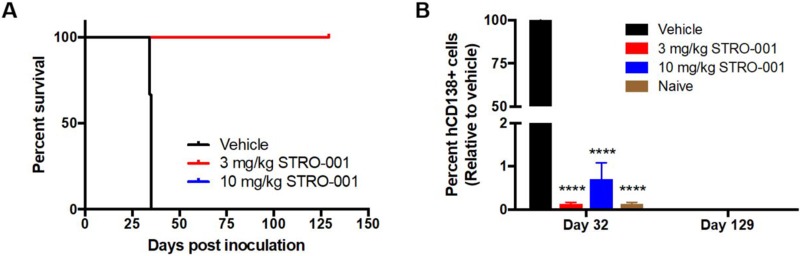
STRO-001 eradicates malignant bone marrow plasma cells and prolongs survival in MM.1S myeloma model NSG mice were inoculated with CD74-expressing MM.1S multiple myeloma cells. Treatment was started at day 11 and repeated once a week for 3 weeks with 3 mg/kg STRO-001 or 10 mg/kg STRO-001. Naïve control mice were not inoculated with tumor cells and did not receive treatment. (**A**) Kaplan-Meier survival curves in response to treatment. Note that 3 mg/kg STRO-001 is superimposed over 10 mg/kg STRO-001. (**B**) Quantification of hCD138+ cells in the bone marrow depicted as percent relative to vehicle control (set as 100% maximum) on days 32 and 129. Graph is shown as average values ± SEM.

The cytotoxic activity of STRO-001 was also tested in another MM cell line model, MM.1S, which primarily homes to the murine BM after intravenous implantation. Tumor challenged mice were treated with either vehicle, 3 or 10 mg/kg STRO-001 starting on day 11 post-tumor inoculation. Animals treated with 3 weekly doses of 3 mg/kg or 10 mg/kg STRO-001 showed no weight loss and 100% survived for longer than 4 months with no sign of disease (Figure 7A). Tumor burden in the BM assessed on day 32 showed that 3 mg/kg or 10 mg/kg STRO-001 significantly diminished tumor burden compared with vehicle control (Figure 7B). At end of study (day 129), STRO-001-treated mice were disease-free and presented negligible levels of hCD138+ cells as observed in naïve control (Figure 7B).

**Figure 8:**
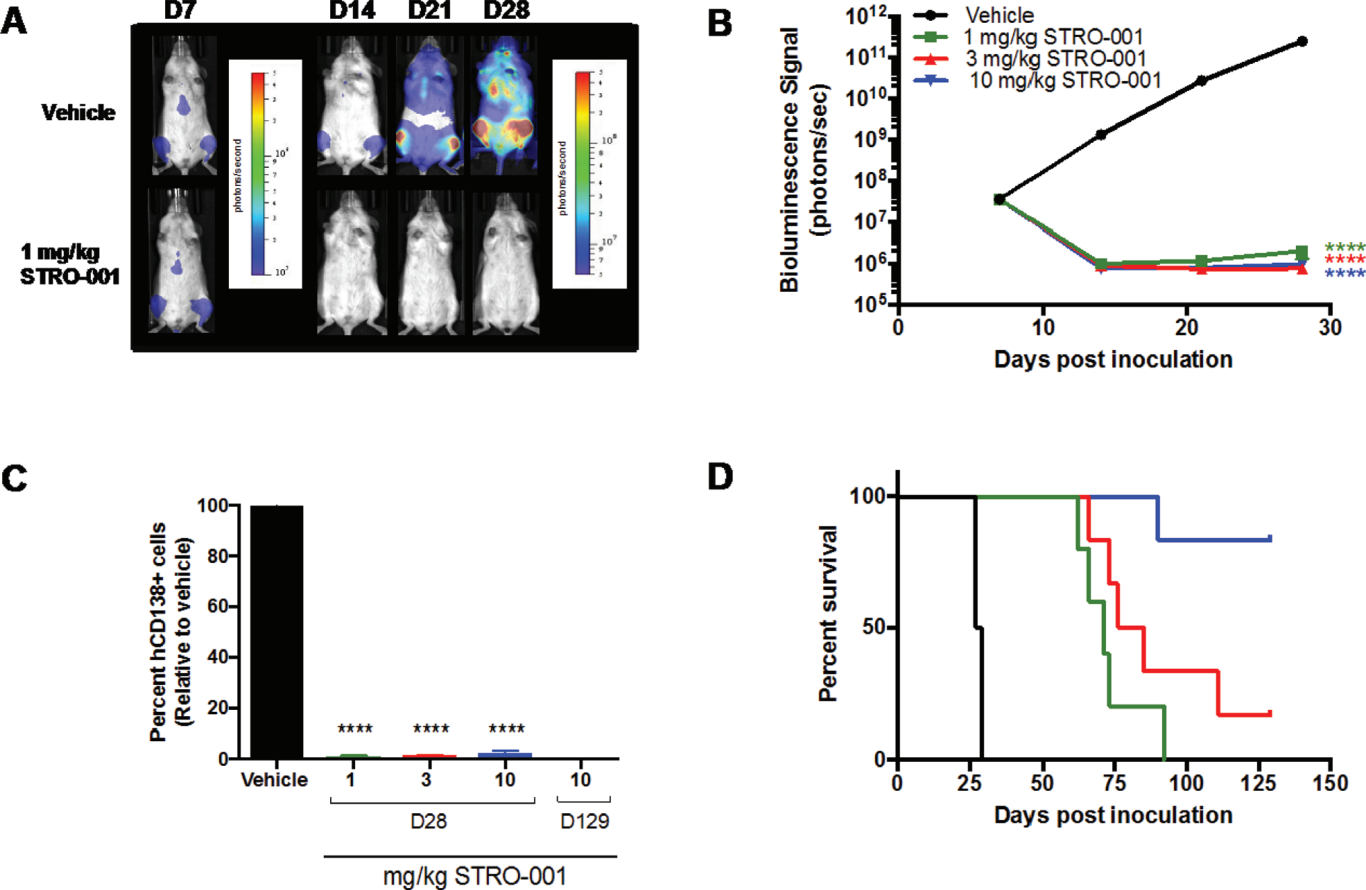
Antitumor activity of STRO-001 on MM.1s model assessed with bioluminescence imaging NSG mice inoculated with MM.1S-luc cells received a single dose of vehicle, 1, 3, or 10 mg/kg STRO-001 at 7 days post-inoculation. (**A**) Representative series of *in vivo* bioluminescence images showing MM.1S-luc cells in the bone marrow of live mice treated with vehicle or 1 mg/kg STRO-001 at different time points. Different color bar scales were used for day 7 (1 × 10^5^ to 5 × 10^6^) and days 14, 21 and 28 (5 × 10^6^ to 5 × 10^8^). Similar response was observed with 3 and 10 mg/kg STRO-001 (data not shown). (**B**) Quantification of bioluminescence in live mice treated with vehicle, 1 mg/kg STRO-001, 3 mg/kg STRO-001 and 10 mg/kg STRO-001. (**C**) Quantification of hCD138+ cells in the bone marrow depicted as percent relative to vehicle control (set as 100% maximum) on days 28 and 129. (**D**) Kaplan–Meier survival curves in response to treatment. Graphs are shown as average values ± SEM.

Next, the dose-response relationship of STRO-001 was evaluated in MM.1S cells engineered to express a bioluminescent reporter luciferase (MM.1S-luc) to allow real-time monitoring of disease progression and tumor burden. On day 7 post-tumor inoculation, tumor-bearing mice were treated with a single dose of 1, 3 or 10 mg/kg of STRO-001 or vehicle control. Bioluminescence imaging (BLI) was utilized to evaluate response to treatment using weekly imaging on days 7, 14, 21 and 28. Engraftment of MM.1S-luc cells was observed on day 7 (approximately 3 × 10^7^ photons/second) and increasing BLI signal reaching a maximum average flux of 2 × 10^11^ photons/second on day 28 was observed in vehicle-treated mice (Figure 8A and 8B). A single dose of STRO-001 at any of the 3 dose levels significantly decreased BLI to approximately 1 × 10^6^ flux and suppressed tumor growth until day 28 compared with vehicle control (Figure 8A and 8B). Analysis by flow cytometry on day 28 confirmed the presence of hCD138+ cells in the BM in vehicle treated tumors and a significant reduction in tumor burden in all STRO-001 treated groups relative to vehicle control (Figure 8C). Furthermore, all doses of STRO-001 significantly prolonged survival (Figure 8D). A single dose of 1 or 3 mg/kg STRO-001 resulted in a mean survival of 71 and 81 days, respectively. A single dose of 10 mg/kg STRO-001 resulted in survival of 5/6 animals that remained tumor-free until day 129 based on assessment of hCD138+ cells in the BM on day 129 (Figures 8C and 8D).

### Pharmacodynamic targeting of B cells by STRO-001 and safety in cynomolgus monkey

STRO-001 binds with similar and high affinity to human and cynomolgus monkey CD74 but does not bind to rat or mouse CD74 (data on file, Sutro Biopharma); cynomolgus monkey is a pharmacologically relevant species in which to investigate a STRO-001 nonclinical toxicity profile. STRO-001 was tolerated at 1, 3, and 10 mg/kg, but not at 30 mg/kg, when intravenously administered to cynomolgus monkeys once every 2 weeks for 2 consecutive doses. Drug-related mortality or unscheduled termination in moribund condition occurred in 2 animals administered 30 mg/kg approximately 2 weeks after the second dose.

At ≥10 mg/kg (scheduled and unscheduled necropsies), dose-related toxicity findings were present primarily in the hematopoietic/lymphoid tissues (femur and sternum bone marrow hypocellularity, mandibular and mesenteric lymph node germinal center necrosis, and thymic lymphocytic depletion). These changes generally correlated with reversible and cyclical decreases in hematology parameters (neutropenia, thrombocytopenia, and anemia) with complete or near-complete recovery by day 43. Bone marrow toxicity and the above hematological findings were considered dose-limiting toxicities at 30 mg/kg and resulted in lack of tolerability and secondary bacterial infections in some tissues (data on file, Sutro Biopharma).

**Figure 9:**
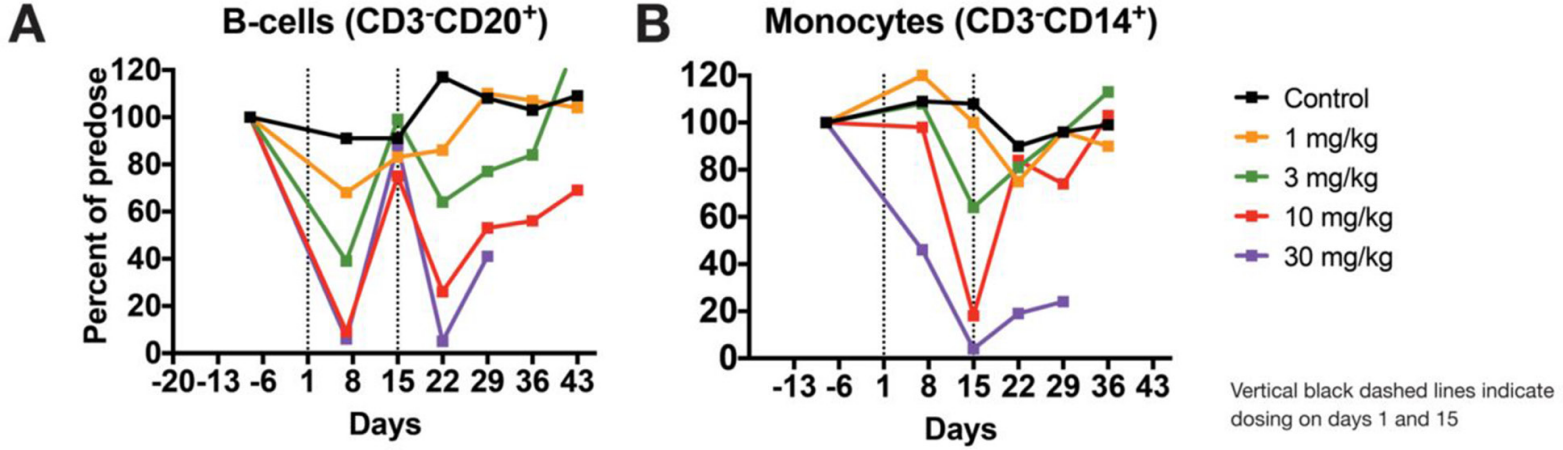
STRO-001 induces dose-responsive ablation of B-cells in cynomolgus monkeys STRO-001 or vehicle control were intravenously administered to cynomolgus monkeys as described herein, and blood was collected at several time points for immunophenotyping analysis. (**A**) Compared with vehicle control, STRO-001 showed dose-dependent and transient decreases in peripheral B-cell levels, with nadirs occurring one week after each dose. (**B**) Similarly, monocyte levels decreased in a dose-dependent manner and reached a nadir at 2 weeks following the first dose. For both B cells and monocytes, these changes showed full reversibility or evidence of reversibility to vehicle control or pre-dose levels by day 43 at tolerated doses (≤10 mg/kg). Data are shown as mean cell levels (percent CD3-CD20+ or CD3^−^CD14^+^) normalized to pre-dose baseline levels ± SEM.

Immunophenotyping analysis in peripheral blood revealed STRO-001-related decreases in absolute and/or mean relative values of peripheral blood B-cell (CD3^−^CD20^+^) and monocyte (CD3^−^CD14^+^) populations at ≥1 mg/kg. These changes in B cells and monocytes were dose dependent, reached statistical significance (*p* < 0.005; ANOVA and Dunnett's test) relative to vehicle control (first dose cycle ≥3 mg/kg), and partially or completely resolved by day 43 at ≤10 mg/kg based on tolerability (Figure 9).

## DISCUSSION

In the quest to more selectively deliver cytotoxins to tumor cells, ADCs are increasingly emerging as agents of interest. Although conceptually they hold the promise of coupling the exquisite specificity of antibody/antigen binding to the subnanomolar potency of antimitotic or DNA-cleaving toxins, experience has uncovered several hurdles that must be overcome for their clinical adoption.

STRO-001 was designed to overcome several known problems previously encountered by ADCs. The antibody portion of the ADC, SP7219, is an optimized anti-CD74 aglycosylated human IgG1 produced in a cell-free expression system. A non-natural amino acid, *para*-azidomethyl-L-phenylalanine is incorporated at a site on each heavy chain that was selected to confer the highest *in vivo* stability for the conjugate. A DBCO-PEG-linked maytansinoid payload, designed to avoid premature cleavage in serum [26], is site-specifically conjugated to SP7219 through copper-free click chemistry [27]. Once catabolized, the linker-payload has limited cell permeability to minimize both export from target cells [28] and “bystander effect” toxicity. In animal studies, ADCs prepared through site-specific methods have demonstrated improved stability and *in vivo* properties relative to the corresponding ADCs prepared through conventional stochastic methods [29], which consist of mixtures of components having distinct PK, efficacy, and safety profiles [30]. Accordingly, STRO-001, a site-specific, predominantly single-species ADC with a fixed drug-antibody ratio of 2, was used to explore the *in vitro* and *in vivo* effects of CD74 targeting in MM.

For new experimental ADCs, the selection of an appropriate antigen that is highly expressed in tumor cells with minimal expression elsewhere is a basic requirement. In the present study, independent, blinded pathology review of bone marrow core biopsies from patients with MM revealed surface CD74 expression in 11/12 and 24/24 samples in newly diagnosed and R/R disease, respectively. The results confirm that CD74 expression is maintained upon disease progression, suggesting that anti-CD74 therapy should be relevant in most cases of newly diagnosed as well as R/R MM.

The ubiquitous detection of CD74 mRNA transcripts in CD138+ enriched plasma cells from all 892 patients in the CoMMpass study is consistent with the detection of CD74 in 35/36 bone marrow biopsies from patients with MM at UCSF. Akin to the heterogeneity of CD74 expression in bone marrow biopsy samples from patients with MM, levels of CD74 surface expression varied considerably in the 6 MM cell lines tested, with 3 of the cell lines (U266B1, MM.1S, and OPM-2) below the lower limit of quantitation. Although STRO-001 displayed a trend towards higher potency with higher surface expression, potent cell killing was still noted for 2 of the 3 cell lines with minimal CD74 surface expression (U266B1 and MM.1S). This likely reflects intracellular delivery driven by the combination of surface copy number and internalization rate, which is known to be particularly high for CD74 [31]. Rapid internalization and quick turnover may thus allow for cells expressing as few as several thousand CD74 receptors to be affected. STRO-001 binds to CD74-expressing cells and effectively inhibits their growth at low nanomolar concentrations, a result with clinical relevance due to the near-ubiquitous CD74 expression found in patient samples in both newly diagnosed and R/R MM. In addition, its activity in cell lines with minimal CD74 expression raises the possibility that efficacy may not entirely depend on IHC-determined CD74 levels in the clinic.

The *in vivo* efficacy of STRO-001 was demonstrated in the ARP-1 and MM.1S xenograft models. In the former, repeat dosing at 3 mg/kg prolonged survival, and significantly reduced tumor burden in bone marrow, kidneys, and ovaries. In the MM.1S model, where myeloma grows primarily in bone marrow, treatment with STRO-001 at 3 mg/kg or 10 mg/kg resulted in no weight loss and 100% survival for at least 4 months, with no signs of disease and negligible levels of hCD138+ cells relative to vehicle control. Thus, repeat dosing of STRO-001 at 3 mg/kg or 10 mg/kg completely abrogated tumors in these models. Dose response was studied using MM.1S cells engineered to express a bioluminescent reporter (MM.1S-luc). BLI revealed that a single dose of STRO-001 at 1, 3 or 10 mg/kg significantly suppressed tumor growth. Survival relative to control was increased in a dose-dependent manner. A single 10 mg/kg dose completely abrogated tumor burden and increased survival 4 months after treatment initiation. In contrast to milatuzumab, which is a glycosylated, CHO-derived monoclonal antibody, STRO-001 produced by ExpressCF+ lacks glycosylation and associated Fc-effector function [24]. The corresponding unconjugated antibody, SP7219, has no independent cytotoxic effect (Figure 5B). Indeed, we have previously reported that SP7219 had no effects on survival or tumor growth when dosed at 10 mg/kg IV every 3 days in the CAG and ANBL-6 xenograft models of myeloma [32]. Similarly, SP7219 was devoid of activity in B-NHL (WSU-DLCL2 and OCI-LY10 DLBCL xenograft) models [33]. All antitumor effects of STRO-001 in the present study must therefore depend on its delivery of the cytotoxic warhead.

Two catabolites have been identified for STRO-001: the major active catabolite SC246 and the minor inactive catabolite SC401. Both have been shown to be much less cytotoxic than a free maytansine benchmark as well as STRO-001 itself. For SC246, this is likely due to its predicted cell impermeability while SC401 lacks a structural feature (C3 ester linkage) known to be important for activity [34]. Regardless of cause, decreased cytotoxicity of these catabolites may lead to an improved safety profile by diminishing non-target-associated toxicity.

Finally, STRO-001 showed a favorable pharmacodynamic profile and an acceptable safety profile in cynomolgus monkeys. Evidence of on-target pharmacology was shown by B-cell and monocyte depletion at all doses and was reversible at all but the highest dose tested. To corroborate these findings, we have confirmed CD74 expression in peripheral B cells and monocytes as previously shown [11] in human and monkey blood (data on file, Sutro BioPharma). From a therapeutic perspective, these data are encouraging as STRO-001 is intended to also target rapidly dividing CD74-positive cells in hematological malignancies in a similar manner to normal B cells and monocytes in monkeys. The STRO-001 nonclinical safety profile consisted of bone marrow hypocellularity and hematology findings, noted predominantly at 10 and 30 mg/kg, with evidence of reversibility at the tolerated dose of 10 mg/kg. From a clinical perspective, these toxicities can be readily monitored in patients with B-cell malignancies during phase 1 dose escalation. In light of these encouraging results in MM, and with evidence of its effects in xenograft models of B-NHL (manuscript in preparation), as well as favorable results in additional preclinical safety studies [35] STRO-001 is currently being clinically investigated in a phase 1 study of patients with MM and B-cell malignancies (NCT03424603).

## MATERIALS AND METHODS

### Generation of STRO-001

The antibody SP7219 was expressed by XpressCF+™ as previously described [26, 36]. Briefly, the cell-free extract was added to a premix containing other additives. The final protein synthesis reaction consisted of 30% cell extract, 1 mM pAMF, 5μM pAMF tRNA synthetase, 2 mM glutathione disulfide, 2 mM amino acids (except 0.5 mM for tyrosine and phenylalanine), and 10 μg/mL plasmid DNA encoding SP7219 heavy chain and light chain polypeptides. An amber stop codon at position F404 of the heavy chain sequence allowed for the incorporation of pAMF using an orthogonal tRNA and tRNA synthetase, as described [26]. Cell-free reactions were initiated by the addition of plasmid DNA and incubation at 30°C for 12 hours in a stirred-tank reactor. The antibody was purified by conventional methods under aseptic conditions. Briefly, Protein A resin was used to affinity purify the antibody, followed by additional purification steps using Capto™ Adhere (GE Healthcare Life Sciences, Pittsburgh, PA) and Ceramic Hydroxapatite Chromatography (Bio-Rad, Hercules, CA) resins. The ADC STRO-001 (Figure 1) was generated by the conjugation of SP7219 with the linker-warhead SC236 through SPAAC, also known as copper-free click chemistry [37]. Briefly, SC236 (DBCO-linker-maytansinoid; Supplementary Appendix, SC236 Synthesis), formulated at 5 mM in DMSO, was added to purified SP7219 at a 1.25:1 molar ratio to the number of sites of conjugation (each antibody contains 2 sites of conjugation, one for each heavy chain strand) and incubated overnight at room temperature. Any unconjugated SC236 was subsequently removed by tangential flow filtration. DAR was determined by reverse phase HPLC as previously described [38].

### MM cell lines and reagents

MC/CAR cells were purchased in 2013 from ATCC (Manassas, VA), MM.1S and U266B cells were purchased from ATCC in 2016. OPM2 cells were purchased from The Leibniz Institute DSMZ in 2013 (Braunschweig, Germany). ARP-1 and ARD cells were obtained from Dr. Jonathan J. Keats in 2016 (University of Arkansas for Medical Sciences/Translational Genomics Research Institute, Phoenix, AZ). All cell lines were maintained in RPMI, high glucose medium (Corning, Corning, NY) supplemented with 20% heat-inactivated fetal bovine serum (Thermo Scientific, Grand Island, NY), 2mM GlutaMAX (Thermo Scientific), and 1× penicillin/streptomycin (Corning).

Cells used in the experiments described below were passaged fewer than 5 times since receipt from the vendors. MM.1S and U266B1 cells tested negative for Mycoplasma pulmonis on June 13, 2016; ARP-1, ARD and MC/CAR cells tested negative for Mycoplasma pulmonis on November 30, 2015. All cell lines were authenticated by short tandem repeat (STR) profiling at ATCC in November 2017. Two cell lines (ARD and ARP-1) were not part of the ATCC or DSMZ STR database. STR analysis of late passage of ARD and ARP-1 cells matched early passage cells obtained from Dr. Jonathan J. Keats.

### Animals

Female CB17 SCID mice 9-weeks old, approximately 22 grams, were obtained from Charles River Laboratories (San Diego, CA), and female NOD/SCID/IL-2Rγ^−/−^ (NSG) mice (8-weeks old, approximately 22–25 grams) were obtained from The Jackson Laboratory (Sacramento, CA). A study with female cynomolgus monkeys, approximately 2 to 4 years old and weighing 2–5 kg, was conducted at Covance Inc. (Madison, WI). All animal studies were performed per their Institutional Animal Care and Use Committee-approved guidelines and protocols.

### IHC staining of MM patient samples

Review of records and use of patient samples were performed under a University of California, San Francisco (UCSF) Committee for Human Research-approved protocol. Paraffin-embedded tissue blocks from the UCSF Medical Center were identified and reviewed following a search in the UCSF Department of Pathology database. Blocks were processed by routine methods, and 4 μm sections were obtained. Detection was performed using the DABMap™ kit for biotinylated antibodies on a Ventana Discovery Ultra, an automated IHC/ISH research slide staining system (Ventana Medical Systems, Tucson, AZ). SP-010094, a biotinylated form of SP7219 anti-CD74 antibody was used at a 1:900 dilution. SP7219 antibody was biotinylated using EZ-Link NHS-PEG4-Biotin (Pierce Cat# 21329) per manufacturer's instructions. Conjugation conditions were performed at room temperature in PBS, using a 50-fold molar excess of biotin-reagent to antibody. Biotinylated antibody was qualified by enzyme-linked immunosorbent assay and subsequently evaluated for IHC at Wax-It Histology Services Inc. (Vancouver, Canada) with appropriate positive and negative controls (Supplementary Appendix).

Plasma cells were identified based on morphology and paired hCD138+ staining. CD74 expression in the plasma cells was measured as follows: 0, no staining; 1+, weak staining; 2+, moderate staining; 3+, strong staining. CD74 expression was evaluated by two pathologists (N.G. and K.W.), who were blinded to the clinical history and pathology results.

### Determination of CD74 transcript expression in patient plasma cells

Gene expression data from CD138+ enriched plasma cells from patients with newly diagnosed MM was downloaded from the Multiple Myeloma Research Foundation (MMRF) CoMMpass study version IA13 [25]. Processed data in TPM from paired-end RNA-seq was used for analysis.

### Determination of ABC of MM cell lines

To measure cell surface ABC, Quantum™ Simply Cellular^®^ anti-human IgG beads (Bangs Laboratories, Inc., Fishers, IN) were used per the manufacturer's description. Briefly, beads were washed separately and re-suspended in fluorescence-activated cell sorting (FACS) buffer (DPBS buffer supplemented with 1% bovine serum albumin, 0.05% sodium azide). Cells were harvested, re-suspended in FACS buffer and blocked with human Fc block buffer (BD, Franklin Lakes, NJ) for 10 minutes on ice. 100 nM anti-CD74 antibody (SP7219) conjugated to DBCO-Alexa647 (Thermo Scientific) was then added into beads or cells and incubated for 60 minutes. Beads and cells were washed twice with ice-cold FACS buffer and analyzed using the BD FACSCanto^TM^ system. Unstained cells were used as negative controls. FACS data were analyzed by FlowJo^®^ software (FlowJo, LLC, Ashland, OR). Geometric MFI of each population of the beads was used to plot a linear standard (MFI versus ABC) using QuickCal^®^ software (Bangs Laboratories, Inc.). ABC of each cell line was interpolated from the standard curve based on the geometric MFI of the cells.

### ADC cell killing assay in MM cell lines

Cytotoxicity effects in MM cell lines were measured with a cell proliferation assay. A total of 12,500 cells in a volume of 25 μL were seeded in a 384-well flat bottom white polystyrene plate on the day of assay. ADCs were formulated at 2x starting concentration in cell culture medium and filtered through MultiScreen HTS 96-Well Filter Plates (MilliporeSigma, Billerica, MA). Filter-sterilized samples were serially diluted (1:3) under sterile conditions and 25 μL per well of the diluted sample was added onto cells. Plates were cultured at 37°C in a CO_2_ incubator for 72 hours. For cell viability measurements, 30 μL of CellTiter-Glo^®^ reagent (Promega Corp, Madison, WI) was added into each well, and plates processed per product instructions. Luminescence was measured on an ENVISION^®^ plate reader (PerkinElmer, Waltham, MA). Relative luminescence readings were converted to percentage viability using untreated cells as controls. Data were fitted with non-linear regression analysis, using log (inhibitor) versus response, variable slope, 4-parameter fit using Prism (GraphPad Software, Inc., La Jolla, CA). Data were expressed as percent relative cell viability versus dose of ADC in nM.

### Multiple myeloma disseminated xenograft models

For the ARP-1 model, 9-week old CB17 SCID female mice approximately 22 grams were pre-treated with 0.4 mg/mouse fludarabine and 2 mg/mouse cyclophosphamide via intraperitoneal (IP) injection 3 days prior to tumor cell inoculation via tail vein with 10 × 10^6^ ARP-1 cells. For MM.1S or MM.1S-luciferase (MM.1S-luc) models, 8-week old NSG female mice approximately 22–25 g were inoculated with 5 × 10^6^ cells (passage 3–5) via tail vein. On the same day, animals were randomized (*n* = 5–8 per group) and treatment was initiated 7–14 days post-cell inoculation and was administered by intravenous injection. In the ARP-1 and MM.1S study, naïve animals which were not inoculated with tumor cells and did not receive treatment were included as controls. Animals were monitored at least twice a week for body weight changes and onset of MM clinical signs including changes in posture, fur, gait, and mobility resulting from hind limb paralysis. Kaplan–Meier curves depict animal survival characterized by substantial body weight change (BWC; defined as greater than 9% gain for ARP-1 model or 20% loss for MM.1S model) or moribundity. BWC was calculated relative to weight on the first day of treatment using the following formula:

((W_current_ -W_initial_)/W_initial_)^*^100 where W_intial_ is the weight on the first day of treatment. All results were reproduced in at least 2 independent studies, and representative data from one experiment is shown.

### Assessment of tumor burden by flow cytometry or *in vivo* BLI

To assess BM tumor burden by flow cytometry, cells from mouse femur and tibia were pooled (due to limited sample size) and stained with anti-hCD138 Alexa Fluor 647 antibody (clone MI15, BD Biosciences, San Jose, CA) per the manufacturer's protocol. Direct immunofluorescence flow cytometric analysis was performed using an LSRII flow cytometer and FACSDiva™ Software (BD Biosciences). Data was analyzed using FlowJo software. The percentage of hCD138+ cells is depicted as percentage maximum of vehicle-control group.

To assess tumor burden pre- and post-therapy, mice bearing MM.1S-luc cells were imaged weekly for 4 weeks starting on the first day of treatment (day 7). Animals were anesthetized with 1%-2% isoflurane gas and injected with 150 mg/kg D-luciferin (IP) for *in vivo* BLI performed using an IVIS^®^ Spectrum (Caliper Life Sciences, Hopkinton, MA). Bioluminescence was monitored on days 7, 14, 21 and 28; images were analyzed using MATLAB R2015a software (MathWorks, Natick, MA). Whole body fixed-volume regions of interest (ROI) were placed on prone and supine images for each individual animal and summed together to estimate whole body tumor burden. Total flux (photons/sec) was calculated and exported for all ROIs to facilitate analyses between groups.

### Dose-ranging exploratory toxicology and pharmacodynamics study in cynomolgus monkeys

Nineteen experimentally naïve female monkeys were randomly assigned to 5 groups. Group consisted of 5 animals each in groups 1 and 5, and 3 animals each in groups 2 through 4. On days 1 and 15, animals were intravenously administered vehicle (10 mM Na-Citrate and 9% Sucrose, pH 6.0; group 1) or STRO-001 at 1, 3, 10, and 30 mg/kg (groups 2, 3, 4, and 5, respectively) at a dose volume of 5 mL/kg. All groups were monitored twice daily for mortality, clinical abnormalities, and food consumption over a 6-week observation period. Blood was collected at pre-dose and at several time points during the study for clinical pathology, toxicokinetic, anti-drug antibodies, and immunophenotyping analyses (helper T cells, cytotoxic T cells, B cells, NK cells, and monocytes). A subset of animals was euthanized and necropsied on day 16 (3 animals each from groups 1 and 5) and day 43 (remaining animals in groups 1 and 4). Based on tolerability, gross and microscopic anatomic pathology evaluations were conducted in several organs/tissues at scheduled and unscheduled terminations in groups 1, 4, and 5. All animals in groups 2 and 3 were returned to the colony.

### Statistical analysis

Percentage of hCD138+ cells, mean survival, and tissue weights were analyzed using a one-way analysis of variance (ANOVA) with Dunnett's multiple comparison test (Prism software version 6). BLI signal was analyzed using two-way ANOVA with Tukey's multiple comparison test. A probability of less than 5% (*p* < 0.05) was considered significant. Annotations for *p*-value are as follows: ^*^= *p* < 0.05; ^**^= *p* < 0.01; ^***^= *p* < 0.001; ^****^= *p* < 0.0001.

## SUPPLEMENTARY MATERIALS APPENDIX





## Abbreviations

ADC: antibody-drug conjugate
BLI: bioluminescence imaging
BM: bone marrow
BWC: body weight change
CoMMpass: Relating Clinical Outcomes in Multiple Myeloma to Personal Assessment of Genetic Profile
DAR: drug-antibody ratio
DBCO: dibenzocyclooctyne
DMSO: dimethylsulfoxide
FACS: fluorescence-activated cell sorting
HPLC: high performance liquid chromatography
IHC: immunohistochemistry
IP: intraperitoneal
MFI: mean fluorescence intensity
MM: multiple myeloma
MMRF: Multiple Myeloma Research Foundation
ROI: regions of interest
R/R: relapsed/refractory
SPAAC: strain-promoted azide-alkyne cycloaddition
STR: short tandem repeats
TPM: transcripts per million
