# Supplementary material for: Targeting CD74 in multiple myeloma with the novel, site-specific antibody-drug conjugate STRO-001
Source: Oncotarget. 2018 Dec 28;9(102):37700–14. doi: 10.18632/oncotarget.26491 (PMC6340874; doi:10.18632/oncotarget.26491)
Supplement: Supplementary file 1 [file oncotarget-09-37700-s001.pdf]

Targeting CD74 in multiple myeloma with the novel, site-specific antibody-drug conjugate STRO-001

SUPPLEMENTARY MATERIALS

Supplementary Appendix 1: Targeting CD74 in multiple myeloma with the novel, site-specific antibody-drug conjugate STRO-001. See Supplementary_Appendix
