# Supplementary material for: Targeting CD74 in multiple myeloma with the novel, site-specific antibody-drug conjugate STRO-001
Source: Oncotarget. 2018 Dec 28;9(102):37700–14. doi: 10.18632/oncotarget.26491 (PMC6340874; doi:10.18632/oncotarget.26491)
Supplement: Supplementary file 2 [file oncotarget-09-37700-s002.docx]

**Targeting CD74 in multiple myeloma with the novel, site-specific antibody-drug conjugate STRO-001**

**SUPPLEMENTARY MATERIALS**

**SC236 Synthesis**

The synthetic route to **SC236** is described in Figure 1

Figure 1

***tert*-butyl 1-hydroxy-3,6,9,12-tetraoxapentadecan-15-oate (1)**

To a solution of tetraethylene glycol (100 g, 515 mol) in anhydrous THF (300.0 mL) was added NaH (60% dispersion in mineral oil, 210 mg, 5.25 mmol) and *t*-butyl acrylate (26 mL, 0.18 mol). The resulting solution was stirred for 20 hours at room temperature. The solvent was removed under reduced pressure. The residue was purified by flash column chromatography (silica gel, 100-200 grade, petroleum ether/EtOAc=1/1Rf=0.2) to afford **compound 1** as a colorless oil (32.9 g, 57%). MS (ESI) *m/z* 340.2 [M+NH_4_]: [M+Na]345.2

***tert*-butyl 1-(tosyloxy)-3,6,9,12-tetraoxapentadecan-15-oate (2)**

To a solution of compound **1** (4.0 g, 12.4 mmol) and 6.6 mL (37.2 mmol) of DIEA in 50 mL of anhydrous DCM at 0℃ (ice-bath), was added 3.31 g of p-tolunenesulfonyl chloride (17.4 mmole) in one lot. The reaction mixture was stirred at room temperature for 2 days. The reaction mixture was extracted with ethyl acetate (100 mL). The organic layer was combined and washed with 5% citric acid (50 mL), water (50 mL), saturated brine (50 mL), dried over anhydrous sodium sulfate, filtered and concentrated *in vacuo*. The recovered residue 4.1 g (HPLC purity 90%) was used in the next step without further **purification.**

MS (ESI) *m/z* [M+Na]499.2

**tert-butyl 1-azido-3,6,9,12-tetraoxapentadecan-15-oate (3)**

To a solution of compound **2** (8.2 g, 17.2 mmole) in ethanol (60 mL) was added sodium azide (2.24 g, 34.4 mmole). The mixture thus obtained was refluxed overnight, allowed to cool and concentrated *in vacuo*. The recovered crude material treated with water (100 mL) and extracted with ethyl acetate (150 mL). The organic layer was washed with water (100 mL), saturated brine (100 mL), dried over anhydrous sodium sulfate, filtered and concentrated *in vacuo*. The recovered residue was purified by flash column chromatography (silica gel, 100-200 mesh, 30 mm x 450 mm, petroleum ether/EtOAc=3/1Rf=0.2) to afford **compound 3** as a light yellow oil (4.1 g).

***tert*-butyl 1-amino-3,6,9,12-tetraoxapentadecan-15-oate (4)**

**Compound 3** (3.0 g, 8.0 mmol) was dissolved in ethanol (20 mL) and 300 mg of 10 percent Pd/C was added. The system was evacuated under vacuum and placed under 1 atm of hydrogen gas via balloon with vigorous stirring, and repeated 4 times to ensure a pure hydrogen atmosphere. The reaction was then stirred overnight at room temperature. Thin layer chromatography showed that the reaction was complete after 16 hours. The crude reaction was passed through a pad of Celite® rinsing with ethyl acetate. The solvent was removed under reduced pressure to afford a light yellow oil, 2.2 g, which was used in the next step without any further purification.

**ter*t*-butyl1,1,1-trifluoro-2-oxo-6,9,12,15-tetraoxa-3-azaoctadecan-18-oat (5)**

To a solution of compound **4** (7.8 g) in methanol (90 mL) was added triethylamine（3.37 g, 33 mmole, 1.5 Eq) and ethyl trifluoroacetate (33 mmole, 1.5 Eq) at 0℃. The reaction mixture was stirred at room temperature overnight, and concentrated *in vacuo*. The recovered crude material was treated with water (100 mL) and extracted with ethyl acetate (100 mL), dried over anhydrous sodium sulfate, filtered and concentrated *in vacuo*. The residue was purified by flash column chromatography to afford compound **6** (7.2 g) ^1^H NMR (CDCl_3_) δ 7.86 (br, 1H); 3.72(t, 2H); 3.59-3.69 (m, 14H). 3.53-3.59 (m, 2H); 2.51 (t, 2H); 1.45(s, 9H).

**5,8,11,14-tetraoxa-2-azaheptadecan-17-oic acid (6)**

To a solution of compound **5** (7.2g, 15.6 mmole, 1 Eq) and 6 mL of anhydrous THF and 3.3g of CH_3_I (23.2 mmole, 1.5 Eq) at 0℃, was added 900 mg of NaH (60% pure in oil, 23 mmole, 1.5 Eq) in small portions, over a period of 10 minutes. After stirring for 15 minutes at 0℃, and then 1 hour at room temperature, the reaction mixture was concentrated to dryness under reduced pressure. The resulting recovered crude material was diluted with 50 mL of THF and 80 mL of water. 3.0 g of LiOH (71.5 mmole, 4.5 Eq) was then added to the reaction mixture, which was then stirred at room temperature for 12 hours. The stirred reaction mixture was then treated with 1N HCl to adjust to pH=6-7. The mixture was then concentrated *in vacuo* to afford crude compound **6** (4.5 g) which was used in the next step without any further purification. MS (ESI) *m/z* [M+H]280.2

**1-(9H-fluoren-9-yl)-4-methyl-3-oxo-2,7,10,13,16-pentaoxa-4-azanonadecan-19-oic acid**

**(7)**

Compound **6** (4.5 g) was slowly added over a period of 5 minutes to Na_2_CO_3_ in 1,4- dioxane at 0℃. Upon completion of addition, the reaction mixture was stirred at room temperature overnight. The reaction mixture was extracted with ethyl acetate (40 mL x 3), adjusted to pH=2-3 by the addition of 1N HCl to make aqueous pH=2-3, and extracted with ethyl acetate (50 mL x 2). The organic layer was washed with water (50 mL), saturated brine (50 mL), dried over anhydrous sodium sulfate, filtered and concentrated *in vacuo*. The residue was purified by flash column chromatography to afford **compound 7 as** a light yellow oil (5.2g) MS (ESI) *m/z* [M+H]502.2

**Fmoc-N-methylamino tetra PEG- DBCO diamide (8)**

HBTU (4.8 g, 12.5 mmol) was added to a solution of PEG-acid **7** (4.8 g, 9.6 mmol) and diisopropylethylamine (3.75 g, 28.8 mmol) in CH_2_Cl_2_ (AR, 100 mL) at room temperature and stirred for 5 minutes. A solution of DBCO-amine (Click Chemistry Tools A-103, 2.86 g, 10.1 mmol) in CH_2_Cl_2_ (25 mL) was added dropwise over a period of 10 minutes. Upon completion of addition, the reaction mixture was stirred overnight at room temperature. Water (100 mL) was added to the reaction mixture. This solution was extracted with CH_2_Cl_2_ (3 × 75 mL). The combined organic solution was washed with saturated brine (2 × 50 mL), and dried over anhydrous Na_2_SO_4_, filtered and the solvent was removed under reduced pressure. The resulting residue was purified by column chromatography (DCM:MeOH=50:1~20:1) on silica gel (200~300 mesh) to yield the desired product (6.5 g, 90% yield).

**N-methylamino tetra PEG- DBCO diamide (9)**

Fmoc-tetra PEG-DBCO **8** (1.0 g, 1.3 mmol) was added to a solution of Et_2_NH (5 mL) in CH_2_Cl_2_ (AR, 10 mL) and stirred overnight at room temperature. The solvent was removed under reduced pressure. 30 mL water was added. This solution was extracted with CH_2_Cl_2_ (3 × 20 mL), washed with saturated brine (3 × 50 mL), dried over anhydrous Na_2_SO_4_, filtered, and the solvent was removed under reduced pressure. The resulting residue was purified by column chromatography (DCM: MeOH=30:1~5:1) on silica gel (200~300 mesh) to yield the product (550 mg, 78% yield). MS *m/z* 538.2 [M+H]^+^

**DBCO N-methylamino diamide tetra PEG carboxylic acid (10)**

Glutaric anhydride (128 mg, 1.1 mmol) in anhydrous THF (10 mL) was added dropwise using a syringe over a period of 10 minutes to a solution of **9** (550 mg, 1.0 mmol) in anhydrous THF (5 mL). Upon completion of addition, the reaction mixture was stirred overnight at room temperature. The solvent was removed under reduced pressure. The resulting residue (quant) was purified by column chromatography (DCM:MeOH=20:1) on silica gel (200~300 mesh) to yield the product (480 mg, 72% yield). MS *m/z* 653.3 [M+H]^+^ 676.2 [M+Na]^+^


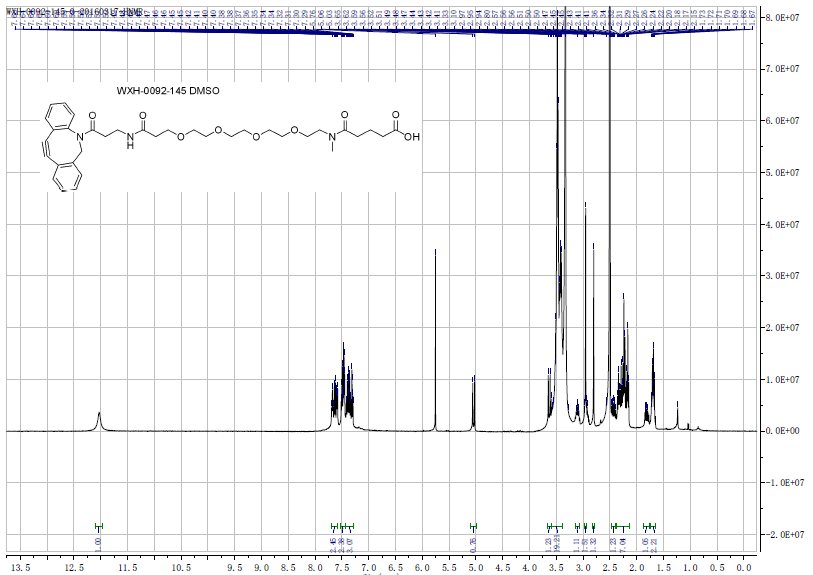


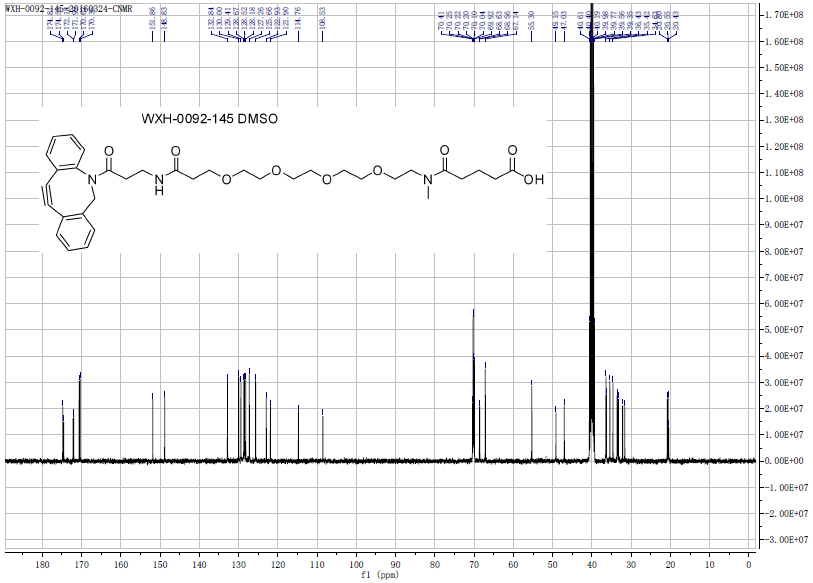


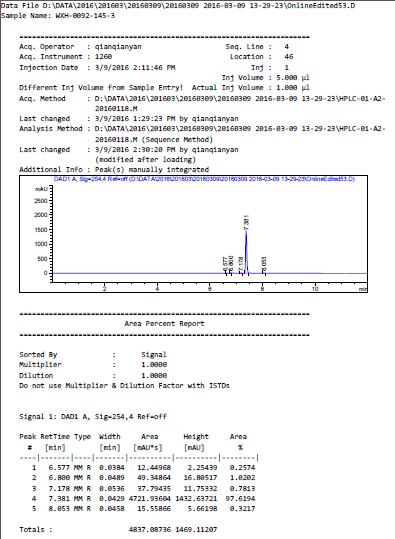


MS:


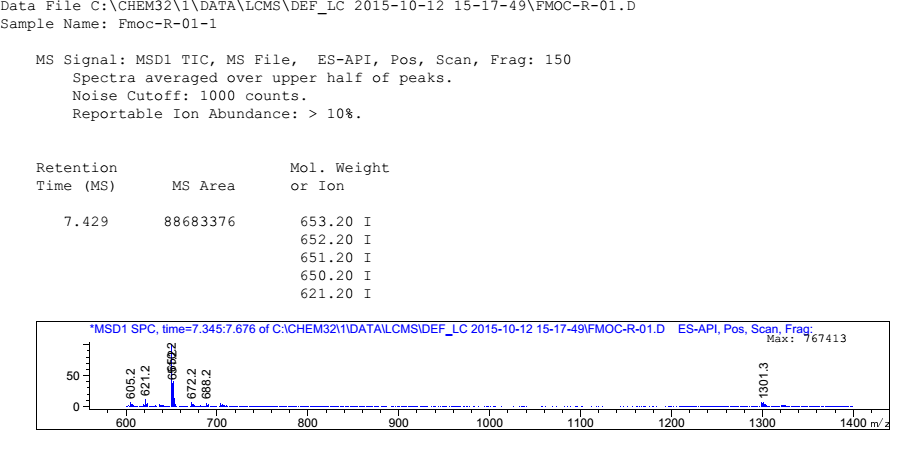


**(S)-3,4-dimethyloxazolidine-2,5-dione (11)**

To a solution of N-(*t*-butoxycarbonyl)-N-methylalanine (2 g, 9.83 mmole) in anhydrous methylene chloride (25 mL) at 0^o^C, was added, under nitrogen, 1.2 Eq (11.80 mmole) of phosphorus trichloride. The reaction mixture was stirred for 2 hours at 0^o^C. The solvent was removed under reduced pressure, and the residue washed with carbon tetrachloride (3 x 20 mL) affording (S)-3,4-dimethyloxazolidine-2,5-dione **11** (1.01 g, 80% yield).

^1^H NMR of **(S)-3,4-dimethyloxazolidine-2,5-dione**


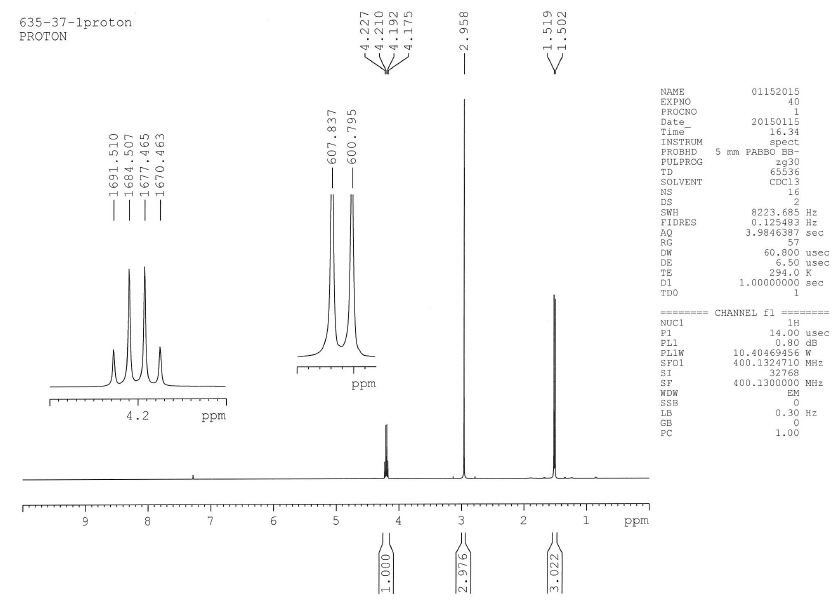


^13^C NMR of **(S)-3,4-dimethyloxazolidine-2,5-dione**


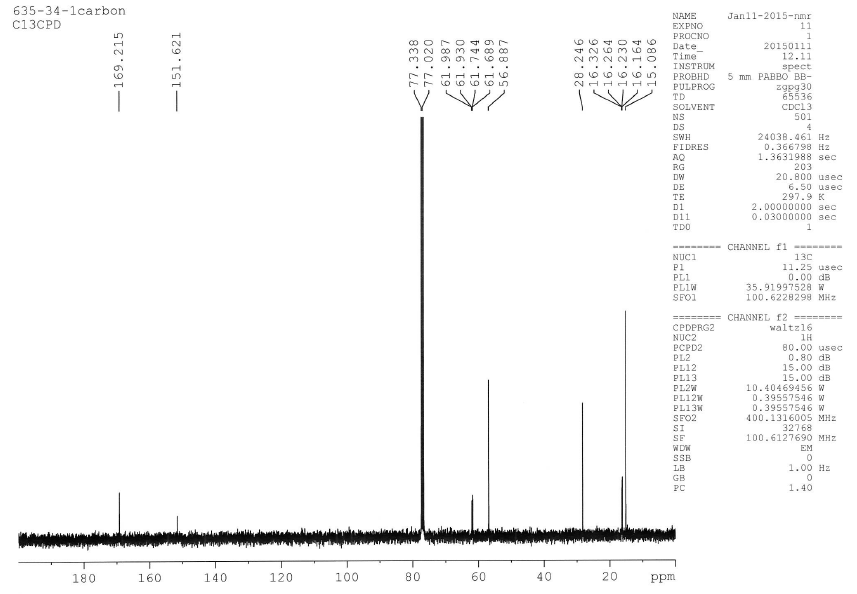


(**14S,16S,32S,33S,2R,4S,10Z,12E,14R)-86-chloro-14-hydroxy-85,14-dimethoxy-33,2,7,10-tetramethyl-12,6-dioxo-7-aza-1(6,4)-oxazinana-3(2,3)-oxirana-8(1,3)-benzenacyclotetradecaphane-10,12-dien-4-yl methyl-L-alaninate (12)**

Maytansinol (ACES Pharma, 150 mg, 0.27 mmol) and (S)-3,4-dimethyloxazolidine-2,5-dione **11**

(120 mg, 0.093 mmol) were dissolved in 6 mL anhydrous DMF. The solution was stirred under an argon atmosphere as zinc bis[bis(trimethylsilyl)amide] (450 mg, 0.12 mmol) was added dropwise over the course of 5-10 minutes, during which time the reaction mixture temperature remained almost unchanged. Upon completion of addition, the reaction mixture was stirred for 6 hours, and analyzed via analytical HPLC (C18 50 mm x 4.6 mm 3 micron). Analysis showed 35% unreacted maytansinol. To the reaction mixture was added 40 mL of 1:1 w/w NaHCO_3_: NaCl solution and extracted with ethyl acetate (15mL x 3). The organic phase was washed with water (20 mL) and saturated brine (20 mL), dried with anhydrous Na_2_SO_4_, filtered, and concentrated under reduced pressure (water bath temperature was 40℃) to give 190 mg of the crude product which is used in the next step, without further purification.

**SC236**

The crude product **12** (190 mg) was dissolved in 30 mL of anhydrous dichloromethane and cooled to 0^o^C. Compound **10** (400 mg, 0.6142 mmole, 4 Eq) and EDCI.HCl (120 mg, 0.6259 mmole, 4 Eq) were added at 0℃. The reaction mixture was stirred for 2 hours, warming to room temperature (25^o^C). The reaction mixture was treated with aqueous 1% NaHCO_3_ (40 mL). The organic layers were washed with water (20 mL) and saturated brine (20 mL), dried over anhydrous sodium sulfate, filtered, and concentrated under reduced pressure. The resulting residue was taken up in a minimum volume of dichloromethane (approximately 2 mL) and purified by column chromatography on silica gel (MeOH : DCM=3:97 to 10:90). The appropriate fractions were combined and concentrated at reduced pressure. The resulting residue was purified by HPLC using a preparative Diazem cyano HPLC column (50 mm x 250 mm, 10 micron Diazem CN column; loading with 2 mL EA) that was equilibrated in a mixture of 2-propanol:hexanes and ethyl acetate (60:16)=0-50%, v/v), as one injection. The desired product was observed via HPLC at a retention time of 17.1 min and the other isomer (epimer at the N-methylalanine α carbon) at 19.6 min. The appropriate fractions containing the desired product were evaporated to give **SC236** as an off-white solid 110 mg (31% over two step)

LCMS : 1284[M+H]+; 1306[M+Na]+;1265[M-H_2_O]+.

**Spectral data for final SC236**

**HPLC**

**LC-MS: (Agilent MSD, G-1946D)**

**Additional experimental data for the synthesis**


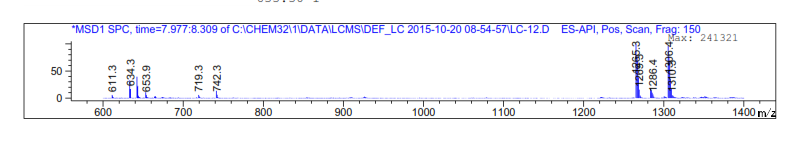


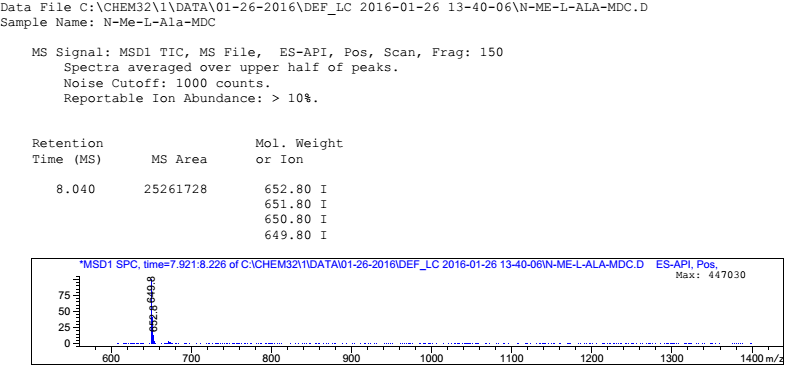


^1^H-NMR(400MHz, d6 DMSO)


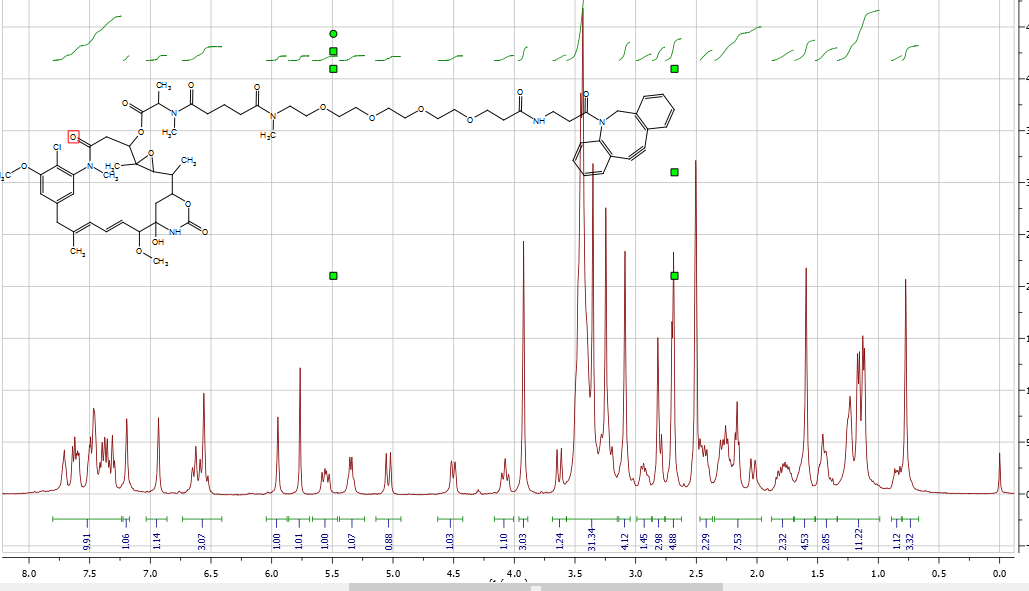


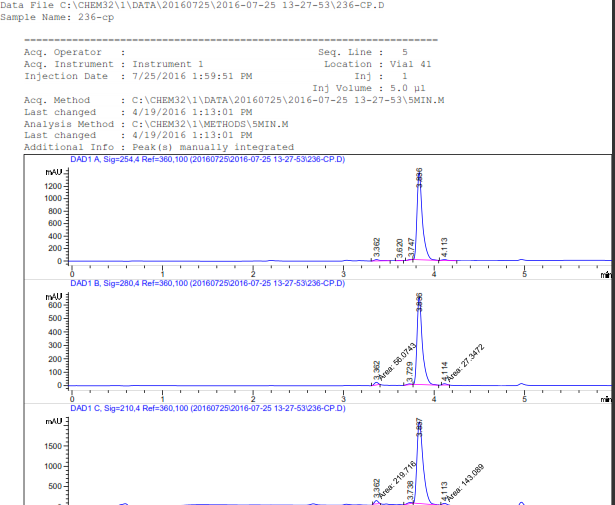


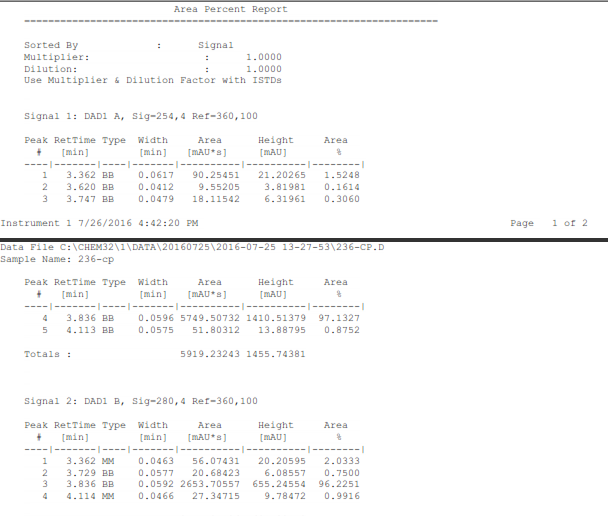


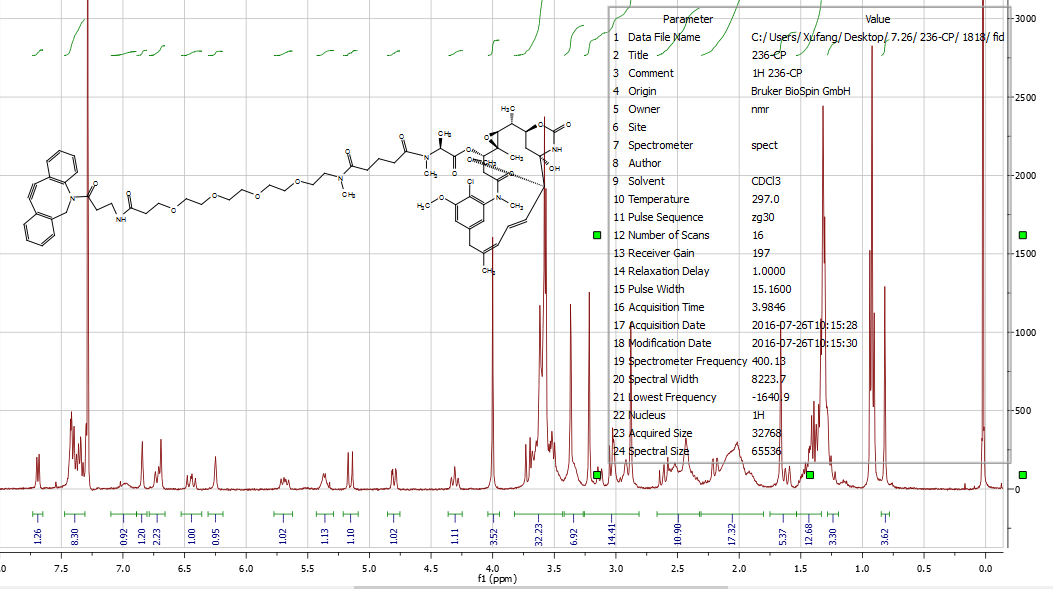


High Resolution Mass Spectrometry.


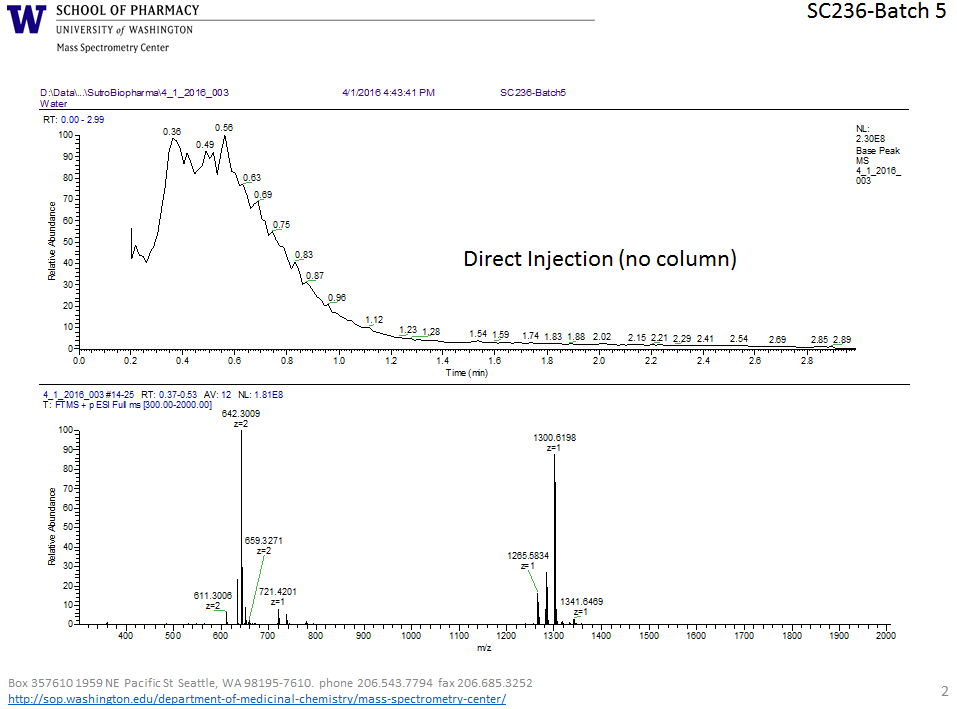

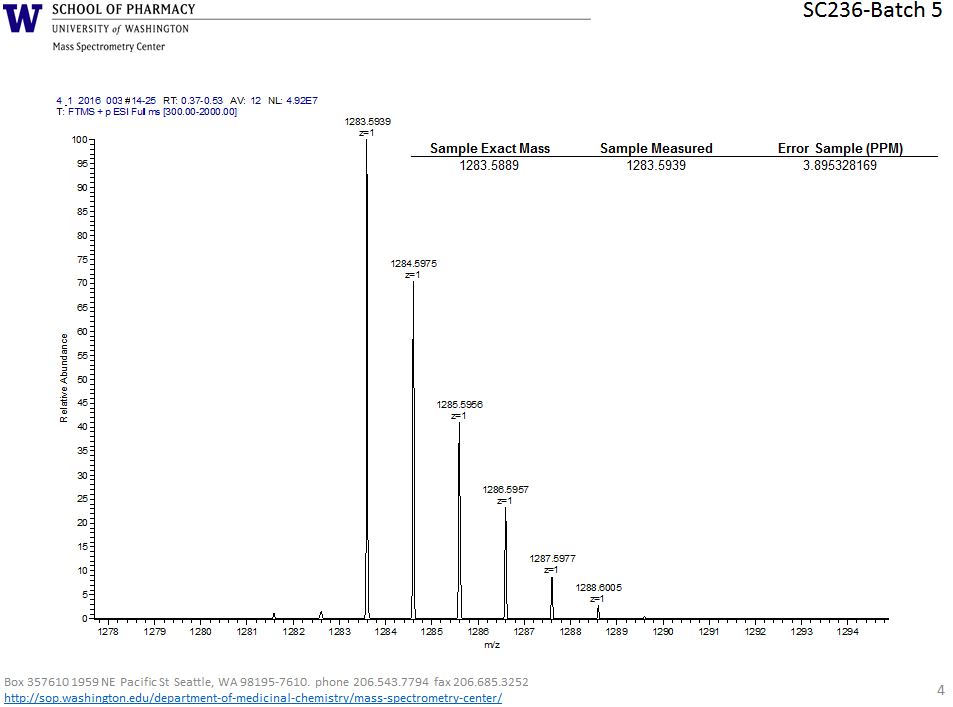


**IHC Method Validation**

The biotinylated anti-human CD74 monoclonal antibody (Biotin-SP7219, SP-010094) was validated for IHC staining on microarrays containing cell lines and human tissues using the Biocare intelliPATH™ auto-stainer (Biocare Medical, Pacheco, CA). The conditions chosen were: 1.25 µg/ml with pH 6.2 heat induced epitope retrieval buffer. Stained slides were digitized with a TissueScope^TM^ LE whole slide scanner (Huron Digital Pathology, St. Jacobs, ON, Canada) at 20x magnification. For confirmation, glass slides were examined on an upright bright-field microscope. Photographs of individual tissues were captured with Huron Viewer software.

For validation, cell lines positive for CD74 expression included CHO-CD74, SuDHL-6 and Raji cells, while negative control cell lines used were CHO, NCI-H929 and OPM-2 (**Supplementary Figure 1**). Normal human tissue negative for CD74 expression (liver and muscle) and positive for CD74 expression (spleen and tonsil) were also stained using the same method (**Supplementary Figure 2**). As seen in these figures the cell lines and tissues showed expected staining for CD74.

**Supplementary Figure 1.** IHC staining for CD74 in multiple myeloma cell lines.

**Supplementary Figure 2.** IHC staining for CD74 in human tissues.
